# The Pólya-Szegö Principle and the Anisotropic Convex Lorentz-Sobolev Inequality

**DOI:** 10.1155/2014/875245

**Published:** 2014-07-15

**Authors:** Shuai Liu, Binwu He

**Affiliations:** Department of Mathematics, Shanghai University, Shanghai 200444, China

## Abstract

An anisotropic convex Lorentz-Sobolev inequality is established, which extends Ludwig, Xiao, and Zhang's result to any norm from Euclidean norm, and the geometric analogue of this inequality is given. In addition, it implies that the (anisotropic) Pólya-Szegö principle is shown.

## 1. Introduction

The classical Pólya-Szegö principle (see, e.g., [1, 2]) states that for *p* ≥ 1 the inequality

(1) $$\begin{array}{c} \int_{R^{n}}{\left|\nabla f\right|}^{p}dx\geq \int_{R^{n}}{\left|\nabla f^{⋆}\right|}^{p}dx \end{array}$$

holds for every *f* ∈ *C*_0_^*∞*^(*R* ^*n*^), where *C*_0_^*∞*^(*R* ^*n*^) denotes the set of functions on *R* ^*n*^ that are smooth and have compact support and |·| is the standard Euclidean norm. Here *f* ^⋆^ denotes the Schwarz symmetrization of *f*, that is, a function whose level sets have the same measure as the level sets of *f* and are dilates of the Euclidean unit ball *B*. It has important applications to a large class of variational problems in different areas, for example, isoperimetric inequalities, optimal forms of Sobolev inequalities, and sharp a priori estimates of solutions to second-order elliptic or parabolic boundary value problems.

An anisotropic version of the classical Pólya-Szegö principle has been proved in [3], where convex symmetrization of *f* is involved, which states that if *K* is an origin-symmetric compact convex set, then for *p* ≥ 1 the inequality

(2) $$\begin{array}{c} \int_{R^{n}}{||\nabla f||}_{K^{o}}^{p}dx\geq \int_{R^{n}}{||\nabla f^{K}||}_{K^{o}}^{p}dx \end{array}$$

holds for every *f* ∈ *C*_0_^*∞*^(*R* ^*n*^), where ||·||_*K*^*o*^_ is the Minkowski functional of  the polar body of  *K*. Here *f* ^*K*^ denotes the convex symmetrization of *f*, that is, a function whose level sets have the same measure as the level sets of *f* and are dilates of the set *K*. Obviously, (2) reduces to (1) when *K* = *B* (see Section 2 for unexplained notation and terminology).

A new approach to understanding Pólya-Szegö principle was proposed recently by Lutwak et al. [4] and Zhang [5]. Instead of using the classical technique on level sets [*f*]_*t*_, their approach is using the *L* ^*p*^ convexification of level sets 〈*f*〉_*t*_. This technique plays a fundamental role in the newly emerged affine Pólya-Szegö principle (see, e.g., [4–9]). Despite this progress, the study of the Pólya-Szegö principle by using this technique is vacancy. This is the motivation of the present paper. More precisely, we show the Pólya-Szegö principle from the *L* ^*p*^ Brunn-Minkowski theory, different from the known proofs of the Pólya-Szegö principle based on the geometric measure theory (see, e.g., [1–3, 10–16]).

In [17], Ludwig et al. proved the following convex Lorentz-Sobolev inequality (see Theorem  2 in [17]): if *f* ∈ *C*_0_^*∞*^(*R* ^*n*^) and 1 ≤ *p* < *n*, then

(3) $$\begin{array}{c} \int_{R^{n}}{\left|\nabla f\right|}^{p}dx\geq n\kappa_{n}^{p/n}\overset{\infty }{\underset{0}{\int }}V{\left({\langle f\rangle }_{t}\right)}^{\left(n-p\right)/n}dt, \end{array}$$

where *V* denotes the Lebesgue measure on *R* ^*n*^ with *κ*_*n*_ = *V*(*B*) = *π* ^*n*/2^/Γ(1 + *n*/2). This inequality has a geometric analogue, namely,  the following *L* ^*p*^ isoperimetric  inequality:  for 1 < *p* < *n*,

(4) $$\begin{array}{c} S_{p}\left(L\right)\geq n\kappa_{n}^{p/n}V{\left(L\right)}^{\left(n-p\right)/n}, \end{array}$$

where *L* is an origin-symmetric compact convex set in *R* ^*n*^ and *S*_*p*_(*L*) is the *L* ^*p*^ surface area of *L*.

In this paper we establish the following anisotropic convex Lorentz-Sobolev inequality.

**Theorem 1 .**

If *f* ∈ *C*_0_^*∞*^(*R* ^*n*^), 1 ≤ *p* ≠ *n*, and *K* is an origin-symmetric convex body in *R* ^*n*^, then

(5) $$\begin{array}{c} \int_{R^{n}}{||\nabla f||}_{K^{o}}^{p}dx\geq nV{\left(K\right)}^{p/n}\overset{\infty }{\underset{0}{\int }}V{\left({\langle f\rangle }_{t}\right)}^{\left(n-p\right)/n}dt \end{array}$$

with equality if and only if 〈*f*〉_*t*_ is a dilate of *K* for almost every *t* > 0.

Similarly, our inequality (5) has a geometric analogue, namely,  the following  *L* ^*p*^ Minkowski  inequality, for 1 < *p* < *n*,

(6) $$\begin{array}{c} V_{p}\left(L,K\right)\geq V{\left(L\right)}^{\left(n-p\right)/n}V{\left(K\right)}^{p/n}, \end{array}$$

where *L*, *K* are origin-symmetric compact convex sets in *R* ^*n*^ and *V*_*p*_(*L*, *K*) is the *L* ^*p*^ mixed volume of *L*, *K*.

When *L* = *B*, from *S*_*p*_(*L*) = *nV*_*p*_(*L*, *B*), (5) and (6) reduce to (3) and (4), respectively.

It is shown that our inequality (5) implies the anisotropic Pólya-Szegö principle (2) for 1 ≤ *p* ≠ *n* in Theorem 5. Hence it is also true in Euclidean case; that is, (3) implies (1) for 1 ≤ *p* ≠ *n*. The arguments after Theorem 5 yield the fact that the anisotropic Pólya-Szegö principle (2) is still true for *p* = *n* if we use the solution to the even normalized *L* ^*p*^ Minkowski problem.

## 2. Background Material

### 2.1. Elements of the *L* ^*p*^ Brunn-Minkowski Theory

For later reference, we quickly recall in this subsection some background material from the *L* ^*p*^ Brunn-Minkowski theory of convex bodies. This theory has its origin in the work of Firey from the 1960s and has expanded rapidly over the last couple of decades (see, e.g., [4, 8, 18–33]).

A convex body is a compact convex set in *R* ^*n*^ which is throughout assumed to contain the origin in its interior. We denote by *K*_*o*_^*n*^ the space of convex bodies equipped with the Hausdorff metric. Each convex body *K* is uniquely determined by its support function *h*_*K*_ = *h*(*K*, ·) : *R* ^*n*^ → *R* defined by

(7) $$\begin{array}{c} h_{K}\left(x\right)=h\left(K,x\right):=\max\left\{x\cdot y:y\in K\right\}. \end{array}$$

Let ||·||_*K*_ : *R* ^*n*^ → [0, *∞*) denote the Minkowski functional of  *K* ∈ *K*_*o*_^*n*^; that is, ||*x*||_*K*_ = min⁡{*λ* ≥ 0 : *x* ∈ *λK*}.

The polar set *K* ^*o*^ of *K* ∈ *K*_*o*_^*n*^ is the convex body defined by

(8) $$\begin{array}{c} K^{o}=\left\{x\in R^{n}:x\cdot y\leq 1\;\;\forall y\in K\right\}. \end{array}$$

If *K* ∈ *K*_*o*_^*n*^, then it follows from the definitions of support functions and Minkowski functionals, as well as the definition of polar body, that

(9) $$\begin{array}{c} h_{K}\left(\cdot \right)=h\left(K,\cdot \right)={||\cdot ||}_{K^{o}}. \end{array}$$

For *p* ≥ 1,  *K*, *L* ∈ *K*_*o*_^*n*^, the *L* ^*p*^ Minkowski combination *K*+_*p*_ *L* is the convex body defined by

(10) $$\begin{array}{c} h{\left(K{+}_{p}L,\cdot \right)}^{p}=h{\left(K,\cdot \right)}^{p}+h{\left(L,\cdot \right)}^{p}. \end{array}$$

The *L* ^*p*^ mixed volume *V*_*p*_(*K*, *L*) of  *K*, *L* ∈ *K*_*o*_^*n*^ is defined in [25] by

(11) $$\begin{array}{c} V_{p}\left(K,L\right)=\frac{p}{n}\underset{\varepsilon \to 0^{+}}{\lim}\frac{V\left(K{+}_{p}\varepsilon^{1/p}L\right)-V\left(K\right)}{\varepsilon }. \end{array}$$

In particular,

(12) $$\begin{array}{c} V_{p}\left(K,K\right)=V\left(K\right) \end{array}$$

for every convex body *K*.

It was shown in [25] that, for all convex bodies *K*, *L* ∈ *K*_*o*_^*n*^,

(13) $$\begin{array}{c} V_{p}\left(K,L\right)=\frac{1}{n}\int_{S^{n-1}}h_{L}^{p}\left(u\right)dS_{p}\left(K,u\right), \end{array}$$

where *S*_*p*_(*K*, *u*) = *h*_*K*_(*u*)^1−*p*^ *dS*(*K*, *u*) and the measure *S*(*K*, ·) on *S* ^*n*−1^ is the classical surface area measure of *K*. Recall that, for a Borel set *ω* ⊂ *S* ^*n*−1^, *S*(*K*, *ω*) is the (*n* − 1)-dimensional Hausdorff measure of the set of all boundary points of *K* for which there exists a normal vector of *K* belonging to *ω*.

Note that

(14) $$\begin{array}{c} S_{p}\left(tK,\cdot \right)=t^{n-p}S_{p}\left(K,\cdot \right) \end{array}$$

for all *t* > 0 and convex bodies *K*.

### 2.2. The Convex Symmetrization of Functions

Given any measurable function *f* : *R* ^*n*^ → *R* such that *V*({*x* ∈ *R* ^*n*^ : |*f*(*x*)|>*t*}) < *∞* for every *t* > 0, its distribution function *μ*_*f*_ : [0, *∞*)→[0, *∞*] is defined by

(15) $$\begin{array}{c} \mu_{f}\left(t\right)=V\left(\left\{x\in R^{n}:\left|f\left(x\right)\right|>t\right\}\right). \end{array}$$

The decreasing rearrangement f* : [0, *∞*)→[0, *∞*] of *f* is defined by

(16) $$\begin{array}{c} f^{*}\left(s\right)=\inf\left\{t\geq 0:\mu_{f}\left(t\right)\leq s\right\}. \end{array}$$

The Schwarz symmetrization of *f* is the function *f* ^⋆^ : *R* ^*n*^ → [0, *∞*] defined by

(17) $$\begin{array}{c} f^{⋆}\left(x\right)=f^{*}\left(\kappa_{n}{\left|x\right|}^{n}\right), \end{array}$$

where |·| is the standard Euclidean norm.

For an origin-symmetric convex body *K*, the convex symmetrization *f* ^*K*^ of *f* with respect to *K* is defined as follows:

(18) $$\begin{array}{c} f^{K}\left(x\right)=f^{*}\left(\kappa_{n}{||x||}_{\tilde{K}}^{n}\right), \end{array}$$

where ${||x||}_{\tilde{K}}$ is the Minkowski functional of $\tilde{K}$, with $\tilde{K}$ being a dilate of *K* so that $V(\tilde{K})=\kappa_{n}$. Note that *f*,  f*, and *f* ^*K*^ are equimeasurable; that is,

(19) $$\begin{array}{c} \mu_{f}=\mu_{f^{*}}=\mu_{f^{K}}. \end{array}$$

Therefore, we have

(20) $$\begin{array}{c} {||f||}_{\infty }=f^{*}\left(0\right)={||f^{K}||}_{\infty }. \end{array}$$

We will frequently apply Federer's co-area formula (see, e.g., [34, page 258]). We state a version which is sufficient for our purposes: if *f* : *R* ^*n*^ → *R* is Lipschitz and *g* : *R* ^*n*^ → [0, *∞*) is measurable, then, for any Borel set *A*⊆*R*,

(21) $$\begin{array}{c} \int_{f^{-1}(A)\cap \{|\nabla f|>0\}}g\left(x\right)dx=\int_{A}\int_{f^{-1}\{t\}}\frac{g\left(x\right)}{\left|\nabla f\left(x\right)\right|}dH^{n-1}\left(x\right)dt, \end{array}$$

where *H* ^*n*−1^ denotes (*n* − 1)-dimensional Hausdorff measure.

### 2.3. The *L* ^*p*^ Convexification of Level Sets

Suppose *f* ∈ *C*_0_^*∞*^(*R* ^*n*^). For each real  *t* > 0, define the level set

(22) $$\begin{array}{c} {\left[f\right]}_{t}=\left\{x\in R^{n}:\left|f\left(x\right)\right|\geq t\right\}. \end{array}$$

By Sard's theorem, for almost every *t* > 0, the boundary

(23) $$\begin{array}{c} \partial {\left[f\right]}_{t}=\left\{x\in R^{n}:\left|f\left(x\right)\right|=t\right\} \end{array}$$

of [*f*]_*t*_ is a smooth (*n* − 1)-dimensional submanifold of *R* ^*n*^ with everywhere nonzero normal vector ∇*f*(*x*).

Now, we explain the technique called the *L* ^*p*^ convexification of  level sets (see [17] for more details). Let *f* : *U* → *R*, where *U* ⊂ *R* ^*n*^ is open, be locally Lipschitz; let *t* > 0; and suppose ∇*f*(*x*) ≠ 0  for almost everywhere on  ∂[*f*]_*t*_ = {*x* ∈ *U* : |*f*(*x*)| = *t*}. For 1 ≤ *p* ≠ *n*, define the *L* ^*p*^ convexification 〈*f*〉_*t*_ of the level set [*f*]_*t*_ as the unique origin-symmetric convex body such that

(24) $$\begin{array}{c} \int_{S^{n-1}}\varphi \left(u\right)dS_{p}\left({\langle f\rangle }_{t},u\right)=\int_{\partial {\left[f\right]}_{t}}\varphi \left(\nu \left(x\right)\right){\left|\nabla f\right|}^{p-1}dH^{n-1}\left(x\right) \end{array}$$

for all even *φ* ∈ *C*(*S* ^*n*−1^), where *ν*(*x*) = −∇*f*(*x*)/|∇*f*(*x*)|.

Thus, equality (24) holds for almost every *t* > 0 if *f* ∈ *C*_0_^*∞*^(*R* ^*n*^).

## 3. The Anisotropic Convex Lorentz-Sobolev Inequality

The following lemma can be proved in the spirit of [17, 31, 35](e.g., see Lemma  3 in [35]).

**Lemma 2 .**

If *f* ∈ *C*_0_^*∞*^(*R* ^*n*^) and *K*, *L* are origin-symmetric convex bodies, then, for almost every *t* ∈ (0, ||*f*||_*∞*_) and 1 ≤ *p* ≠ *n*, 〈*f* ^*K*^〉_*t*_ is a dilate of *K* and

(25) $$\begin{array}{c} V\left({\langle f^{K}\rangle }_{t}\right)=V\left({\langle f^{L}\rangle }_{t}\right). \end{array}$$

**Proof**

Since *h*_*K*^*o*^_ is Lipschitz (and therefore differentiable almost everywhere) and *h*_*K*^*o*^_(*x*) = 1 on ∂*K*, then, for almost every *x* ∈ ∂*K*,

(26) $$\begin{array}{c} \nu_{K}\left(x\right)=\frac{\nabla h_{K^{o}}\left(x\right)}{\left|\nabla h_{K^{o}}\left(x\right)\right|}, \end{array}$$

where *ν*_*K*_(*x*) is the outer unit normal vector of *K* at the point *x*. Note that *h*_*K*_(∇*h*_*K*^*o*^_(*x*)) = 1, for almost every *x* ∈ *R* ^*n*^; hence we have

(27) $$\begin{array}{c} h_{K}\left(\nu_{K}\left(x\right)\right)=\frac{1}{\left|\nabla h_{K^{o}}\left(x\right)\right|}. \end{array}$$

Since *f* ^*K*^ is Lipschitz, then, for almost every *t* ∈ (0, ||*f*||_*∞*_), the set ∂[*f* ^*K*^]_*t*_ is the boundary of a dilate of *K* with nonvanishing normal ∇*f* ^*K*^. It follows from Sard's theorem that

(28) $$\begin{array}{c} H^{n}\left(\left\{x\in R^{n}:\left|f\left(x\right)\right|=t\right\}\right)=0\;\text{for}\;\;\text{almost}\;\;\text{every}\;\;t>0. \end{array}$$

Hence there exists a unique *s* > 0 such that *t* = f*(*κ*_*n*_ *s* ^*n*^) for almost every *t* ∈ (0, ||*f*||_*∞*_). Indeed, we have *s* = (*μ*_*f*_(*t*)/*κ*_*n*_)^1/*n*^. Then by (24), (18), (9), and the fact that $\nabla h_{{\tilde{K}}^{o}}$ is homogeneous of degree 0 and (27), we obtain that

(29) ∫Sn−1φp(u)dSp(〈fK〉t,u)=∫∂[fK]tφp(ν(x))|∇fK(x)|p−1dHn−1(x)=∫∂[fK]tφp(ν(x))×|(f∗)′(κnhK~o(x)n)nκnhK~o(x)n−1∇hK~o(x)|p−1dHn−1(x)=∫s∂K~φp(ν(x))|(f∗)′(κnsn)nκnsn−1∇hK~o(x)|p−1dHn−1(x)=sn−1(−(f∗)′(κnsn)nκnsn−1)p−1 ×∫∂K~φp(νK~(x))|∇hK~o(x)|p−1dHn−1(x)=(μf(t)κn)(n−1)/n(−(f∗)′(μf(t))nκn(μf(t)κn)(n−1)/n)p−1 ×∫∂K~φp(νK~(x))hK~(νK~(x))1−pdHn−1(x)=np−1κn(p−n)/nμf(t)(n−1)p/n(−μf′(t))1−p ×∫Sn−1φp(u)dSp(K~,u)

for almost every *t* ∈ (0, ||*f*||_*∞*_) and even *φ* ∈ *C*(*S* ^*n*−1^). Thus, the uniqueness of the solution of the even *L*_*p*_ Minkowski problem [25] and (14) implies that

(30) $$\begin{array}{c} {\langle f^{K}\rangle }_{t}=c{\left(f,t\right)}^{1/\left(n-p\right)}\tilde{K}\;\text{for}\;\;\text{almost}\;\;\text{every}\;\;t\in \left(0,{||f||}_{\infty }\right), \end{array}$$

where *c*(*f*, *t*) = *n* ^*p*−1^ *κ*_*n*_^(*p*−*n*)/*n*^ *μ*_*f*_(*t*)^(*n* − 1)*p*/*n*^(−*μ*_*f*_′(*t*))^1−*p*^. Since *f* ^*cK*^ = *f* ^*K*^ for any *c* > 0 and any *K* ∈ *K*_*o*_^*n*^, we have

(31) $$\begin{array}{c} V\left({\langle f^{K}\rangle }_{t}\right)=V\left({\langle f^{L}\rangle }_{t}\right)=c{\left(f,t\right)}^{n/\left(n-p\right)}\kappa_{n} \end{array}$$

for almost every *t* ∈ (0, ||*f*||_*∞*_).

Recall that the *L* ^*p*^ Minkowski inequality [25] states the following.

**Theorem 3 .**

If *p* ≥ 1 and *L*, *K* ∈ *K*_*o*_^*n*^, then

(32) $$\begin{array}{c} V_{p}\left(L,K\right)\geq V{\left(L\right)}^{1-p/n}V{\left(K\right)}^{p/n} \end{array}$$

with equality if and only if  *L*, *K* are dilates when *p* > 1 and if and only if *L*, *K* are homothetic when *p* = 1.

Now, we prove the anisotropic convex Lorentz-Sobolev inequality.

**Proof of Theorem 1.**

Noting that *h*_*K*_(·) = ||·||_*K*^*o*^_, by the co-area formula (21), (24), (13), and (32), we have

(33) ∫RnhK(∇f)pdx=∫0∞∫∂[f]thK(∇f)p1|∇f|dHn−1(x)dt=∫0∞∫∂[f]thK(ν(x))p|∇f|p−1dHn−1(x)dt=∫0∞∫Sn−1hK(u)pdSp(〈f〉t,u)dt=∫0∞nVp(〈f〉t,K)dt≥nV(K)p/n∫0∞V(〈f〉t)(n−p)/ndt,

where *ν*(*x*) = −∇*f*(*x*)/|∇*f*(*x*)| on ∂[*f*]_*t*_ for almost every *t* > 0 and the second equality holds since *K* is an origin-symmetric and the support function of *K* is homogeneous of degree 1.

Equality (5) follows from equality (32) and the fact that 〈*f*〉_*t*_ is origin-symmetric.

It is shown above, Proof of Theorem 1, that the *L* ^*p*^ Minkowski inequality (32) implies inequality (5).

In what follows we will show that the *L* ^*p*^ Minkowski inequality (32) can be easily deduced from the anisotropic convex Lorentz-Sobolev inequality (5) for 1 < *p* < *n* by taking

(34) $$\begin{array}{c} f\left(x\right)=g\left({||x||}_{L}\right),\;\text{where}\;\;g\left(s\right)={\left(1+s^{p/\left(p-1\right)}\right)}^{1-n/p}. \end{array}$$

Indeed, as shown in [17, Lemma 8],

(35) $$\begin{array}{c} {\langle f\rangle }_{t}=c_{p}\left(t\right)L, \end{array}$$

and *c*_*p*_(*t*)^*n*−*p*^ = |*g*′(*s*)|^*p*−1^ *s* ^*n*−1^ with *t* = *g*(*s*). Hence

(36) ∫Rn||∇f||Kopdx∫0∞nVp(〈f〉t,K)dt=∫0∞nVp(cp(t)L,K)dt=nV(L,K)∫0∞cp(t)n−pdt,∫0∞V(〈f〉t)(n−p)/ndt=∫0∞V(cp(t)L)(n−p)/ndt=V(L)(n−p)/n∫0∞cp(t)n−pdt,

where

(37) $$\begin{array}{c} \overset{\infty }{\underset{0}{\int }}c_{p}{\left(t\right)}^{n-p}dt=\frac{{\left(n-p\right)}^{p}}{{\left(p-1\right)}^{p-1}p}B\left(\frac{n-p}{p},\frac{np-n+p}{p}\right). \end{array}$$

## 4. The Pólya-Szegö Principle

The following theorem can be seen as a weak form of the Pólya-Szegö principle (2).

**Theorem 4 .**

If *f* ∈ *C*_0_^*∞*^(*R* ^*n*^) and *K*, *L* are origin-symmetric convex body such that *L* is not a dilate of *K*, then, for 1 ≤ *p* ≠ *n*,

(38) $$\begin{array}{c} \int_{R^{n}}{||\nabla f^{L}||}_{K^{o}}^{p}dx>\int_{R^{\text{n}}}{||\nabla f^{K}||}_{K^{o}}^{p}dx. \end{array}$$

**Proof**

Since 〈*f* ^*L*^〉_*t*_ is a dilate of *L* for almost every *t* ∈ (0, ||*f*||_*∞*_) by Lemma 2, then the *L* ^*p*^ Minkowski inequality (32) between 〈*f* ^*L*^〉_*t*_ and *K* is strict for almost every *t* ∈ (0, ||*f*||_*∞*_). Combined with (25), it follows that

(39) ∫Rn||∇fL||Kopdx=∫0∞nVp(〈fL〉t,K)dt>nV(K)p/n∫0∞V(〈fL〉t)(n−p)/ndt=nV(K)p/n∫0∞V(〈fK〉t)(n−p)/ndt=∫0∞nVp(〈fK〉t,K)dt=∫Rn||∇fK||Kopdx.

We are now in the position to prove the Pólya-Szegö principle (2).

**Theorem 5 .**

Suppose *K* is an origin-symmetric convex bodies in *R* ^*n*^. If *f* ∈ *C*_0_^*∞*^(*R* ^*n*^), 1 ≤ *p* ≠ *n*, then

(40) $$\begin{array}{c} \int_{R^{n}}{||\nabla f||}_{K^{o}}^{p}dx\geq \int_{R^{n}}{||\nabla f^{K}||}_{K^{o}}^{p}dx. \end{array}$$

**Proof**

It was shown in [4, (6.3)] that the following differential inequality holds:

(41) $$\begin{array}{c} V{\left({\langle f\rangle }_{t}\right)}^{\left(n-p\right)/n}\geq n^{p-1}\mu_{f}{\left(t\right)}^{(n-1)p/n}{\left(-\mu_{f}^{\prime }\left(t\right)\right)}^{1-p}. \end{array}$$

Integrating both sides of the inequality gives

(42) ∫0∞V(〈f〉t)(n−p)/ndt ≥np−1∫0∞μf(t)(n−1)p/n(−μf′(t))1−pdt.

Noting that *h*_*K*_(·) = ||·||_*K*^*o*^_ and Combined with (5), we obtain that

(43) ∫RnhK(∇f)pdx ≥npV(K)p/n∫0∞μf(t)(n−1)p/n(−μf′(t))1−pdt.

By the homogeneous of *K* in (43) and (40), we only need to consider *V*(*K*) = *κ*_*n*_. So it is sufficient to prove that

(44) $$\begin{array}{c} \int_{R^{n}}h_{K}{\left(\nabla f^{K}\right)}^{p}dx=n^{p}\kappa_{n}^{p/n}\overset{\infty }{\underset{0}{\int }}\mu_{f}{\left(t\right)}^{(n-1)p/n}{\left(-\mu_{f}^{\prime }\left(t\right)\right)}^{1-p}dt. \end{array}$$

The last equality is shown in [3]. Now, we prove this equality by using Lemma 2. Together with the co-area formula (21), the equality (24), the definition of *c*(*f*, *t*) in Lemma 2, (13), and *V*(*K*) = *κ*_*n*_, we obtain

(45) ∫RnhK(∇fK(x))pdx =∫0∞(∫∂[fK]thK(∇fK(x))p|∇fK(x)|dHn−1(x))dt =∫0∞∫∂[fK]thK(ν(x))p|∇fK|p−1dHn−1(x)dt =∫0∞∫Sn−1hK(u)pdSp(〈fK〉t,u)dt =∫0∞∫Sn−1c(f,t)hK(u)pdSp(K,u)dt =∫0∞c(f,t)dt∫Sn−1hK(u)pdSp(K,u) =nV(K)∫0∞np−1κn(p−n)/nμf(t)(n−1)p/n(−μf′(t))1−pdt =npκnp/n∫0∞μf(t)(n−1)p/n(−μf′(t))1−pdt,

where *ν*(*x*) = −∇*f* ^*K*^(*x*)/|∇*f* ^*K*^(*x*)| on ∂[*f* ^*K*^]_*t*_ for almost every *t* > 0. And the second equality holds since *K* is origin-symmetric and the support function of *K* is homogeneous of degree 1.

Moreover, Theorem 5 can be proved for *p* ≥ 1 by using the solution to the even normalized *L* ^*p*^ Minkowski problem as in [7, 9]. More precisely, suppose *f* ∈ *C*_0_^*∞*^(*R* ^*n*^), for *p* ≥ 1, and define the normalized *L* ^*p*^ convexification $\tilde{{\langle f\rangle }_{t}}$ as the unique origin-symmetric convex body such that

(46) 1V(〈f〉t~)  ∫Sn−1g(u)dSp(〈f〉t~,u) =∫∂[f]tg(ν(x))|∇f|p−1dHn−1(x),

for almost every *t* > 0. By taking slight modifications in the proof of Theorem 1, we obtain

(47) $$\begin{array}{c} \int_{R^{n}}h_{K}{\left(\nabla f\right)}^{p}dx\geq nV{\left(K\right)}^{p/n}\overset{\infty }{\underset{0}{\int }}V{\left(\tilde{{\langle f\rangle }_{t}}\right)}^{-p/n}dt. \end{array}$$

Similar to the proof of Theorem 5, together with the observation in [7, (4.22)] that

(48) $$\begin{array}{c} V{\left(\tilde{{\langle f\rangle }_{t}}\right)}^{-p/n}\geq n^{p-1}\mu_{f}{\left(t\right)}^{(n-1)p/n}{\left(-\mu_{f}^{\prime }\left(t\right)\right)}^{1-p}, \end{array}$$

we also get (43). So Theorem 5 remains true for *p* = *n*.
